# Characterization of anti-drug antibodies and drug-responsive B-lymphocytes in piperacillin hypersensitive patients with cystic fibrosis

**DOI:** 10.1186/2045-7022-4-S3-P113

**Published:** 2014-07-18

**Authors:** Mohammed Amali, Andrew Sullivan, John Farrell, Xaoli Meng, Roz Jenkins, Paul Whitaker, Daniel Peckham, B Kevin Park, Dean J Naisbitt

**Affiliations:** 1 University of Liverpool, MRC Centre for Drug Safety Science, UK; 2 St. James Hospital, Cystic fibrosis Unit, UK

## 

Cystic fibrosis is the most common autosomal recessive condition in Caucasians and recurrent infections lead to a plethora of complications. Repeated courses of high dose intravenous β-lactam antibiotics, such as piperacillin, are employed for the treatment of respiratory exacerbations. Unfortunately, delayed-type hypersensitivity reactions develop in between 26-50% of treated patients. We have recently described the cellular immunological processes that underlie drug-specific response in hypersensitive patients; however, the involvement of the humoral immune system has not been studied. The aim of this study was to quantify piperacillin-specific antibodies in plasma of hypersensitive and tolerant patients and investigate whether B-cells can be stimulated to secrete antibodies in vitro following drug stimulation. Drug-specific antibodies were quantified by ELISA using piperacillin-modified BSA as an antigen. Adducts generated using different drug-protein ratios were used to measure the degree of conjugation that elicits an antibody response. BSA was modified with different β-lactam antibiotics to define structural specificity. Specificity for the piperacillin BSA adduct was confirmed by hapten inhibition. Mass spectrometry was used to characterize the Lys residues modified with piperacillin. B-cells isolated from PBMC were cultured with piperacillin for 5 days and IgG secretion and B-cell activation was measured using ELISpot and flow cytometry (CD19, CD27), respectively. A significantly higher level of anti-piperacillin IgG antibody was detected in plasma of hypersensitive patients when hypersensitive and tolerant patients were compared. Hapten inhibition ELISA confirmed specificity for the piperacillin antigenic determinant. Antibody binding was detected with adducts generated at piperacillin:BSA ratios between 1:1 and 100:1. In contrast, antibody binding was not detectable with penicillin G, amoxicillin or aztreonam BSA conjugates. IgG antibody secretion was also detected from purified B-cells from hypersensitive patients cultured with soluble piperacillin. Drug treatment was associated with increased expression of the B-cell activation marker CD27. These data begin to describe the drug-specific humoral immune response in piperacillin hypersensitive patients with cystic fibrosis. Further work is needed to define the role different antibody subtypes play in the disease pathogenesis.

